# Supplementary material for: Association between Psychopathological Symptoms and Aggression and Selected Biochemical Parameters in Adolescents with Behavioural and Emotional Disturbances
Source: Int J Mol Sci. 2023 Apr 12;24(8):7097. doi: 10.3390/ijms24087097 (PMC10139169; doi:10.3390/ijms24087097)
Supplement: Supplementary file 1 [file ijms-24-07097-s001.zip › ijms-2301497-supplementary.pdf]

Article

Association between psychopathological symptoms and aggression and selected biochemical parameters in adolescents with behavioural and emotional disturbances.

Supplementary Materials:

Table S1. Sociodemographic characteristics of the study group based on data obtained from the case history.

**Data**	**Characteristics**	**n**	**%**
GENDER	girls	82	66.7.
	boys	41	33.3
AGE GROUP	13–14 years	30	24.4
	15–18 years	93	75.6
PLACE OF RESIDENCE	village	31	25.2
	small town	8	6.5
	average-sized town	35	28.5
	big town	49	39.8
FAMILY	functional	16	13.0
	dysfunctional	107	87.0
SIBLINGS	0	18	14.6
	1	45	36.6
	2	22	17.9
	3	13	10.6
	4	12	9.8
	5	5	4.1
	7	1	0.8
	8	7	5.7

PLACE OF RESIDENCE	family home	79	64.2
	foster home	20	16.3
	institutions (including children's home, municipal care centre, emergency shelter)	24.	19.5
ALCOHOL PROBLEM IN THE FAMILY	no	62	50.4
	yes	61	49.6
MENTAL ILLNESS IN THE FAMILY	no	87	70.7
	yes	36	29.3
DEATH IN THE FAMILY (LAST YEAR)	no	118	95.9
	yes	5	4.1
SUICIDE IN THE FAMILY	no	116	94.3
	yes	7	5.7

Table S2. General, clinical characteristics of the study group.

Parameters	Mean ± SD	Median	Q1-Q3
AGE (years)	15.4±1.3	15	15-17
BODY MASS (kg)	61.5±11.6	60	53-68
HEIGHT (m)	1.66±0.08	2	2-2
BMI (kg/m^2)	22.3±4.0	21	19-24
SYSTOLIC PRESSURE (mm Hg)	127.3±12.7	128	120-136
DIASTOLIC PRESSURE (mm Hg)	75.3±10.7	75	68-81
PULSE RATE (beats/minute)	86.6±13.1	87	78-93
BODY TEMPERATURE (°C)	36.8±0.3	37	37-37

HOSPITALIZATION TIME (days)	19.9±11.9	19	10-27
WBC (K/μL)	6.3±1.4	6	5-7
RBC (M/μL)	4.7±0.5	5	4-5
HGB (g/dL)	13.5±1.4	13	12-14
HCT (%)	40.3±3.7	40	37-43
PLT (K/μL)	259.8±64.7	256	218-299
ESR (mm)	9.0±7.1	7	4-11
SODIUM (mmol/l)	140.2±2.3	140	139-142
POTASSIUM (mmol/l)	4.3±0.3	4	4-5
GLUCOSE (mg/dl)	84.3±7.7	84	79-90
CREATININE (mg/dl)	0.6±0.1	1	1-1
ALT (U/l)	20.6±44.0	14	11-18
AST (U/l)	20.7±7.8	18	16-23
CRP (mg/l)	1.2±2.1	0	0-1
TSH (μU/ml)	2.1±1.0	2	1-3
CORTISOL (μg/l)	134.8±68.6	132	88-173
BDNF (pg/ml)	2391±2331	1655	663-3080

SD – standard deviation, Q1-Q3 – lower quartile, upper quartile.

BMI – body mass index, WBC – white blood cell count, RBC – red blood cell count, HGB – haemoglobin concentration, HCT – hematocrit, PLT – platelet count, ESR – erythrocyte sedimentation rate (red blood cell descent rate), ALT – alanine aminotransferase AST – aspartate aminotransferase, CRP – C-reactive protein, TSH – thyrotropin, BDNF – neurotrophic brain-derived factor

Table S3. Characteristics of the study group based on data from psychometric tests.

Trait dimensions	Mean ± SD	Median	Q1-Q3

SOM_SCL	1.0±0.9	1	0-2
OC_SCL	1.4±1.1	1	1-2
SENS_SCL	1.5±1.0	1	1-2
DEP_SCL	1.8±1.1	2	1-2
ANX_SCL	1.3±1.1	1	0-2
ANG_SCL	1.4±1.0	1	1-2
PHO_SCL	0.9±1.0	1	0-2
PAR_SCL	1.4±1.1	1	1-2
PSY_SCL	1.0±0.9	1	0-2
A_BP	21.0±5.8	21	17-25
PA_BP	23.7±8.5	23	17-30
H_BP	25.4±6.8	25	21-31
VA_BP	15.3±4.3	15	12-19
SUM_BP	85.4±20.5	85	73-97

SD – standard deviation, Q1-Q3 – lower quartile, upper quartile.

SCL-90 Questionnaire: SOM_SCL – somatization, OC_SCL – obsessive-compulsive, Sens_SCL – interpersonal sensibility, DEP_SCL – depression, ANX_SCL – anxiety, ANG_SCL – anger-hostility, PHO_SCL – phobic-anxiety, PAR_SCL – paranoid ideation, PSY_SCL – psychoticism. Buss-Perry Questionnaire: A_BP – anger, PA_BP – physical aggression, H_BP – hostility, VA_BP – verbal aggression, SUM_BP – sum.
